# Editorial 2024

**DOI:** 10.1098/rsos.240679

**Published:** 2024-06-12

**Authors:** Wendy Hall

**Affiliations:** ^1^ Web Science Institute, University of Southampton, Southampton SO17 1BJ, UK

It is an exciting time to be writing this Editorial. Wearing my artificial intelligence (AI) hat, I see its theory, practice and regulation developing at a rapid pace. This is my third year as Editor-in-Chief of *Royal Society Open Science*. And it is the tenth anniversary of the journal’s launch.

In AI, there has been remarkable progress in this potentially transformative technology. News stories abound with new applications of generative pre-trained transformers (GPTs), and the growing sophistication of their performance. Much interest has been devoted to OpenAI’s Chat GPT-4 and its abilities to generate human-like text from a large corpus of data, as well as close-to life-like images generated by machines such as DALL E. I have spoken often in the media about the challenges to, for instance, the integrity of the democratic process by AI technologies that generate outputs increasingly indistinguishable from human-derived content, especially in a year in which many countries will be taking to the polls. In this vein, *Royal Society Open Science* worked with the Royal Society’s Policy team and academic experts to commission and publish a piece last year,^1^ which made the point that humans are (or, perhaps more accurately given the pace of change, were) able to detect deepfake content in a minority of cases.

During my tenure as Editor-in-Chief, I have been delighted to see not only a return of submission numbers to the journal to near pre-COVID levels but also a reduction in times to editorial decisions. I hope the former reflects the high esteem and quality with which the journal is viewed by the communities we serve. I know the latter reflects both the hard work of the Editorial Board and the volunteer referees on whom we rely to carefully and swiftly assess works submitted for consideration—thank you to all of you who have served on the Board and as referees, the journal team and I are grateful for your support. I have also enjoyed seeing the continued growth of the Science, Society and Policy section of the journal, and its diversification into a broader range of subjects (at launch in 2021, much content published in the section was—understandably—COVID-focused).

The good news stories about the journal above lead me to our tenth anniversary. The journal published its first papers in September 2014 and has gone from strength to strength since. To date, we have published over 5900 manuscripts, the 2023 Impact Factor is 3.5, and *Royal Society Open Science* regularly features in various media, contributing to papers often receiving high (and growing) Altmetric scores. The objective peer reviewing model we operate—in other words, trying to avoid making assessments and assumptions about the likely future significance of submitted papers—is somewhat vindicated in the fact that our debut papers have almost all (49 out of 52, and two of those are errata) been cited at least once in the intervening 10 years according to the Scopus index. The journal has positioned itself as an early adopter (indeed, first-mover in many cases) of open science features—for instance, Registered Reports and a formal Replication study policy, and more recently, we agreed to be the first journal to welcome content first posted to the Octopus service (in the spirit of openness, I was Chair of the judging panel that awarded Octopus a Royal Society prize in 2018).

As well as reflecting on the journal’s earlier successes—many of which are elucidated in earlier Editorials—our tenth anniversary year provides an opportunity to look ahead. We want you, our readers, to celebrate the tenth anniversary with us. Not only will we be sharing classic content with you afresh but we also want you to get involved. Tell us how the journal has been a positive actor for change in scholarly communications; tell us how the work you published with us has impacted your subsequent career; tell us what you would like the journal to do in the future. Naturally, I have some ideas about the future, but I want the journal to be a collaborative space in the best traditions of open science. To be the best provider to our communities, we need your support and engagement—I invite you to work with us in a number of ways.

Firstly, we are commissioning a collection of papers with an AI policy focus. This is such a fast-moving field, but getting AI policy right is going to be essential for science and society. The collection will build on themes explored at a recent workshop held at the Royal Society on responsible AI governance,^2^ and work with which I am involved as a member of the United Nations Advisory Board on AI. I will be working with Prof Nick Pearce in the Science, Society and Policy section, along with the editorial office and Editorial Board, to develop a curated living collection of papers exploring as many aspects of AI policy as we can. I would like the collection to comprise not only specially commissioned pieces but papers submitted in response to this open call: please get in touch with the journal editorial office if you would like to be involved or offer suggestions.

Secondly, that science has often deliberately or inadvertently excluded individuals and groups is not news, but we want to ensure that we play an active role in correcting historical absences. We are taking steps to encourage wider representation on our Editorial Board and in the diversity of our reviewer pool. From encouraging authors to suggest a representative group of referees when they submit their paper to appointing a more diverse Editorial Board,^3^ we aim to reflect more effectively the fact that science is global. Please work with us to volunteer as referees or Editors, or help us access your networks of aspiring and established scientists who might not think of working with a Royal Society journal: we are open to all.

Finally, we want the science that we publish to be read and shared widely. Sign up for our table of contents alerts and our newsletters to make sure you do not miss the excellent research, reviews and other papers we publish in *Royal Society Open Science*. If you use social media, share our content there, too. In a polarized and complicated world, making sure credible and evidence-backed science is available to all is essential.

No longer in a start-up phase, *Royal Society Open Science* has matured into a well-regarded scientific publication over the last 10 years. I am excited to see what the next 10 years will bring, I hope you will be too.

## Footnotes

^1^
Deepfake detection with and without content warnings (https://royalsocietypublishing.org/doi/10.1098/rsos.231214)

^2^
The Royal Society. The United Nations’ role in International AI Governance [Internet]. 2024 [accessed 2024 April 23]. Available from: https://royalsociety.org/news-resources/publications/2024/un-role-in-international-ai-governance/

^3^

*Royal Society Open Science*. Editorial Board [Internet]. 2024 [accessed 23 April 2024]. Available from: https://royalsocietypublishing.org/rsos/editorial-board

